# Centering equity, diversity, and inclusion in youth digital mental health: findings from a research, policy, and practice knowledge exchange workshop

**DOI:** 10.3389/fdgth.2024.1449129

**Published:** 2024-10-31

**Authors:** Medard Adu, Bilikis Banire, Mya Dockrill, Alzena Ilie, Elizabeth Lappin, Patrick McGrath, Samantha Munro, Kady Myers, Gloria Obuobi-Donkor, Rita Orji, Rebecca Pillai Riddell, Lori Wozney, Victor Yisa

**Affiliations:** ^1^ Department of Psychiatry, Dalhousie University, Halifax, NS, Canada; ^2^ Department of Computer Science, Dalhousie University, Halifax, NS, Canada; ^3^ Department of Psychology, Dalhousie University, Halifax, NS, Canada; ^4^ Maritime SPOR Support Unit, Halifax, NS, Canada; ^5^ Centre for Research in Family Health, Halifax, NS, Canada; ^6^ Department of Psychology, Acadia University, Wolfville, NS, Canada; ^7^ Mental Health and Addictions, Nova Scotia Health, Halifax, NS, Canada; ^8^ Department of Psychology, York University, Toronto, ON, Canada; ^9^ Mental Health and Addictions, IWK Health, Halifax, NS, Canada

**Keywords:** mental health, technology, diversity, equity and inclusion, interdisciplinary, translational science, knowledge translation

## Abstract

**Background:**

Youth mental health service organizations continue to rapidly broaden their use of virtual care and digital mental health interventions as well as leverage artificial intelligence and other technologies to inform care decisions. However, many of these digital services have failed to alleviate persistent mental health disparities among equity-seeking populations and in some instances have exacerbated them. Transdisciplinary and intersectional knowledge exchange is greatly needed to address structural barriers to digital mental health engagement, develop and evaluate interventions with historically underserved communities, and ultimately promote more accessible, useful, and equitable care.

**Methods:**

To that end, the Digital, Inclusive, Virtual, and Equitable Research Training in Mental Health Platform (DIVERT), the Maritime Strategy for Patient Oriented Research (SPOR) SUPPORT (Support for People and Patient-Oriented Research and Trials) Unit and IWK Mental Health Program invited researchers, policymakers, interprofessional mental health practitioners, trainees, computer scientists, health system administrators, community leaders and youth advocates to participate in a knowledge exchange workshop. The workshop aimed to (a) highlight local research and innovation in youth-focused digital mental health services; (b) learn more about current policy and practice issues in inclusive digital mental health for youth in Canada, (c) participate in generating action recommendations to address challenges to inclusive, diverse and equitable digital mental health services, and (d) to synthesize cross-sector feedback to inform future training curriculum, policy, strategic planning and to stimulate new lines of patient-oriented research.

**Results:**

Eleven challenge themes emerged related to white-colonial normativity, lack of cultural humility, inaccessibility and affordability of participating in the digital world, lack of youth and community involvement, risks of too much digital time in youth's lives, and lack of scientific evidence derived from equity-deserving communities. Nine action recommendations focused on diversifying research and development funding, policy and standards, youth and community led promotion, long-term trust-building and collaboration, and needing to callout and advocate against unsafe digital services and processes.

**Conclusion:**

Key policy, training and practice implications are discussed.

## 1 Introduction

There is mass proliferation of digital mental health services for youth involving remote sensing and wearables, chatbots, health and wellness behavior apps, bioinformatics tools, virtual reality, virtual visits, moderated online social therapy, text message services, patient portals, self-directed technologies for diagnosis, treatment, and decision support, and services informed by increasingly complex data analytics and artificial intelligence (AI) (1–6). Yet, innovations continue to be developed and tested with majority populations and their benefits continue to be unevenly distributed among equity-deserving populations (7, 8). There is a long history of technological advances either disproportionately benefiting already privileged groups (9) and even going so far as disadvantaging marginalized groups (10). As a newer field, youth-focused digital mental health draws together transdisciplinary expertise across computer science and health sectors and could play a key role in disrupting these historical patterns.

Although digital health services offer a critical opportunity to improve wellbeing and decrease the burden of mental illness for the global population, its content and methods must represent and reflect the conceptions of wellness and priorities of people in historically underrepresented populations alongside evidence-based models for care (11). Conceptions of mental health and treatment must be understood before digital health services are built and researched, as they play a significant role in shaping health-seeking behaviors, treatment decisions, perceptions of therapeutic experiences, and recovery. Transdisciplinary and intersectional knowledge exchange is greatly needed to understand how underserved and under-represented youth populations conceptualize mental wellness and digital treatment options, as well as what their expectations and motivations are for seeking support through digital modalities (11–13). If this deeper collaborative work is overlooked, then any deployment of technological innovation may fail to result in improved mental health and substance use outcomes for youth most in need (14), or worse, create further inequity or harm.

Health system decision-makers, computer scientists, mental health researchers, and educational leaders in this field need to develop deep understandings of anti-racist, anti-oppressive and intersectionality theories (15) and be willing to examine their own positionality and biases. One example of these gaps is evidenced in a review of mobile mental health app evaluation frameworks developed since 2015, in which only 58% of them considered at least one EDI criterion (16). Those involved in digital mental health services must commit to undertaking disruptive actions to redress entrenched colonial, systemic, and structural inequities in digital mental health service and research spaces that continue to afford unearned advantage to some and oppress others (17).

Foundational to fostering shared action towards more equitable and inclusive digital mental health is shared knowledge. Shared knowledge is enabled by trust, communication, the use of intermediaries and experiential opportunities (18). One venue to capture collective transdisciplinary wisdom in complex environments of rapidly changing knowledge, technical innovation, and practice like digital mental health are knowledge exchange workshops that focus on deliberate dialogue among diverse partners (19). Knowledge exchange events promote dynamic and non-linear communication and relationship-building that can accelerate impactful research and facilitate its application for the benefit of society (20).

## 2 Method

### 2.1 Knowledge exchange workshop

As an extension to local cross-sector digital equity policy work (21) a scan of gaps in digital youth mental health services standards in Canada (1), implementation research within organizations that have youth digital mental health mandates (22, 23), focus groups with youth in the community and findings from local province-wide roll out of digital metal health services (24), this article reports on the findings of the “*Inclusive Child & Youth Mental Health for the Digital Age*” knowledge exchange workshop in Halifax/Kjipuktuk (Kjipuktuk is the Mi’kmaw name meaning “Great Harbour”), Canada in November, 2023. The knowledge exchange workshop was seen as a strategic platform to convene, share insights, foster collaboration and innovation among researchers, policymakers, practitioners, and those with lived experience.

The workshop aimed to (a) highlight local research and innovation in youth-focused digital mental health services; (b) learn more about current policy and practice issues in inclusive digital mental health for youth in Canada, (c) participate in generating recommendations and opportunities to address challenges to inclusive, diverse and equitable digital mental health services, and (d) to synthesize cross-sector feedback to inform training curriculum, policy, strategic planning and to stimulate new lines of patient-oriented research.

### 2.2 Co-host partners

The Digital, Inclusive, Virtual, and Equitable Research Training in Mental Health Platform (DIVERT) (https://divertmentalhealth.ca) is funded through the Canadian Institutes of Health Research (CIHR). DIVERT is a national transectoral (academic, patient and family, industry, health services) and transdisciplinary (psychology, social work, computer science, rehabilitation, medicine, and nursing) training program dedicated to gathering and promoting diverse approaches to how we understand, teach, research and provide mental health to children, youth, and their families. In addition, at its core it sets out to promote practical knowledge about technologies that can facilitate mental health care. DIVERT co-investigators and trainees working on the East Coast of Canada span multiple research institutions and labs working at the intersection of digital technologies and well-being (e.g., Persuasive Computing Lab, PROSIT, Corkum LABS, Centre for Research in Family Health).

The Maritime Strategy for Patient Oriented Research (SPOR) SUPPORT (Support for People and Patient-Oriented Research and Trials) Unit (collectively referred to as the MSSU) (https://mssu.ca/) works across the Maritime provinces (Nova Scotia, New Brunswick and Prince Edward Island) in Canada and is co-funded by the Canadian Institutes of Health Research (CIHR) initiative and individual provinces. The MSSU works as a connector between key sectoral groups by collaborating with patient/citizen partners, government, healthcare organizations, and the research community to ensure diverse perspectives in research (25).

The IWK Mental Health and Addictions Program is part of an academic women and children's teaching hospital in Halifax, NS. It has a complex pediatric mental health and substance use mandate. First, the program links to and from public health and community-based supports (e.g., non-governmental agencies) and primary care providers (e.g., family physicians and nurse practitioners). It also delivers secondary (e.g., community outpatient services) care for the surrounding metropolitan area, and tertiary (e.g., intensive or specialized) care for the whole province (e.g., only inpatient psychiatry unit). Last, is supports quaternary care for select mental health services (e.g., eating disorders, inpatient concurrent substance misuse/mental health disorders) for the broader region of Canadian east coast provinces. The mental health and addictions (MHA) team participates in a provincial governance group working with government and other health organizations to co-design a digital mental health strategy and service options for youth.

### 2.3 Structure

The workshop was organized into five main elements:

#### 2.3.1 Indigenous knowledge sharing

As Indigenous knowledges have historically been silenced by dominant knowledge organization systems and practices, we sought to honour Indigenous knowledges at the event. A Mi’kmaw community member and invaluable shearer of cultural wisdom and multiple ways of knowing facilitated time for attendees to learn about the Mi’kmaw Medicine Wheel and sacred medicines through a hands-on sensory experience and storytelling. The community member also invited interested attendees to participate in a smudging ceremony and prayer of encouragement, inspiration and connection prior to the event.

#### 2.3.2 Keynote

To situate the three main intersecting ideas of the event (youth mental health, digital innovation and equity, diversity, inclusivity, reconciliation, and accessibility an opening keynote address was given by the Nominated Principal Applicant for the DIVERT research team (Dr. Rebecca Pillai Riddell).

#### 2.3.3 Pitch event

Five trainees (ranging from bachelor to post-doctoral stages of training) participated in a rapid one-minute pitch event to expose attendees to concrete examples of what a “digital mental health service” looks like, and how equity-diversity-inclusion (EDI) considerations show up in the design and research undertaken with these services (see Supplementary File 1). All other attendees were invited to act as panel judges and were given a scorecard to rate the pitches on two criteria (a) presentation structure and (b) engagement and clarity.

#### 2.3.4 Discussion of challenges and action recommendations

The core element of the workshop was the formation of groups aimed at facilitating transsectoral/transdisciplinary discourse of key dimensions of digital strategies in child and youth mental health. Studies have consistently demonstrated the effectiveness of group formation methods in workshops, particularly those that prioritize diverse perspectives and intercultural, interdisciplinary collaboration (26). This approach ensures that attendees contribute varied insights, experiences, and expertise, thereby fostering a rich and dynamic environment conducive to meaningful knowledge exchange. Consequently, we incorporated intercultural and interdisciplinary factors into the group formation process, aligning with best practices identified in the literature. Attendees were organized into seven small groups (4–6 members each). Pre-event demographic data was used as a guide to shape maximum variation in group composition including professional disciplines (computer science, nursing, psychology), sectors (government, health systems, university), racially and ethnically minoritized group identity, gender, leadership levels (trainees, emerging, senior), and primary role of attendees (lived experience, advocate, clinician, administrator, researcher, policy-maker). Facilitators at each table were given a listen of four discussion-starter questions with prompts to guide attendees in conversation. For example the first question in the guide: Why aren't digital mental health services for youth and families as inclusive, equitable and diverse as they should be? Included two further prompts: (a) Are there assumptions or bias we make about digital mental health?; and (b) What worries you most about the current state of digital mental health for youth? Following a short break attendees returned to match those challenges with action recommendations. Each table then reported back during a large group discussion to validate and build on each other's ideas. Attendees were encouraged to jot down additional ideas on sticky notes and add to a dedicated wall space.

#### 2.3.5 Panel to showcase future intersections of research, policy, and practice

The workshop closed out with an interactive, audience-guided panel discussion. The 4-member panel was intentionally selected to maximize diversity across disciplines (computer science, psychology, psychiatry), gender, role (policy, clinicians, intervention developer), career stage (trainees, established researchers) and racially and ethnically minoritized group identity.

### 2.4 Steering group and attendees

The Steering Committee members were identified via requests to DIVERT Co-Investigators and IWK MHA leaders seeking trainees or students, in particular from equity-deserving groups. Among the 11-member volunteer group were individuals with lived experience with mental health or substance use challenges, members of racially and ethnically minoritized groups, newcomers to Canada, gender diverse, trainees at various stages of study across multiple professional disciplines (computer science, psychology/neuroscience), representatives from both clinical and policy roles at both provincial health authorities, as well as representation from all three host organizations. A list of potential attendees was generated that bridged local health system leadership, government, youth and caregiver advocacy, EDI advocacy, researchers, innovation leaders, trainees and faculty working on digital mental health projects at the local and national level. A limit of 40 event attendees was determined at outset due to budget and planning constraints.

### 2.5 Pre-event preparation

Facilitators and note-takers for small-group table discussions were recruited and a pre-event meeting was held to provide tips and strategies for facilitating dialogue, prompts to encourage deeper reflection, and to answer any questions. A note-taking sheet and guide were developed and made available to facilitators. Trainees participating in the Pitch Event were given two months’ notice to develop their pitch slides (one to two slides) and were provided criteria on how the pitches would be scored along with tips for preparing a great pitch. At the request of panelists, a series of mock questions were provided two-weeks in advance to help prepare them for the discussion.

### 2.6 Workshop logistics

The 3-hour event was held from 9:00a.m. to 12:00 p.m., November 6th, 2023 on the Dalhousie University campus. We promoted networking interactions (breakfast event prior to meeting, short breaks, dedicated meet-and-greet time for trainees afterwards). A brief pre-workshop survey was used to collect attendee demographic data. Attendees were informed prior to registration and during the event that a de-identified summary of event learnings would be made publicly available. Attendees who were trainees or community members with lived experience received $100 honorariums. As the workshop involved evaluation for program planning rather than research, no ethical approval was necessary based on Article 2.5 of the Tri-Council Policy Statement: Ethical Conduct for Research Involving Humans (27).

### 2.7 Data gathering, analysis and validation

Nine sets of field notes from table facilitators and trainees along with sticky notes from poster boards in the room were digitized. Rapid analysis approaches have the potential to deliver valid, timely findings while taking less time to complete (28) so a rapid analysis of attendee input captured in facilitator notes was undertaken and informed by the Framework Method (29). As an initial step, familiarization with all nine sets of facilitators’ notes required multiple line-by-line close readings after which a paraphrase or label (a “code”) was applied that described what was interpreted to be most important in each line. Keywords, descriptors or technical terms were also underlined as a way to capture emphasis, tone, and urgency. After coding four sets of notes, one author (LW) compared the labels that had been applied and developed a set of initial codes. This preliminary coding framework was then applied to the remaining sets of notes but with flexibility to add a “new” code if something emerged that did not fit any of the existing coding labels. Charting of key themes was aimed at striking a balance between reducing the data on the one hand and retaining the original meanings and “feel” of the facilitator's words on the other. Gradually, characteristics of and differences between the codes were identified and wording of key themes refined iteratively. The process was undertaken separately for key themes related to “challenges” and “actions”. The positionality of the primary coder (e.g., white, cis-gender academic) necessitated further discussion and meaning-making with a broader group of voices. To review the credibility of the interpretation and improve trustworthiness, an initial draft of themes was shared and discussed with eleven members of the authorship group, a number of whom were facilitators at the event and the rest attendees themselves. Their direct experience at the event was vital to the interpretive process of coding. The broader coding review group was racially, ethnically, age and gender diverse with expertise across disciplines, organizational affiliations and career stage. Members had opportunities to provide and reflect on each other's written comments on the preliminary coding as well as engage in large-group or individual discussions about the codes. For example, a virtual data-analysis meeting with the authorship team was held to review early synthesis efforts, explore rival interpretations, refine theme structures and work towards consensus around key implications. Through this process codes were in some instances modified, regrouped, or renamed into the final version. This member-checking and validation process helped ensure accuracy and resonance with attendees’ experiences (30).

An online post-event evaluation survey was co-developed to align with other DIVERT and MSSU regional meeting evaluation processes. The critical evaluation allowed attendees to provide insights on the event's structure, content, and overall experience. The four-question survey was emailed to all attendees the morning following the event. Descriptive statistics were used to summarize results.

## 3 Results

### 3.1 Attendees

**Figure 1:**
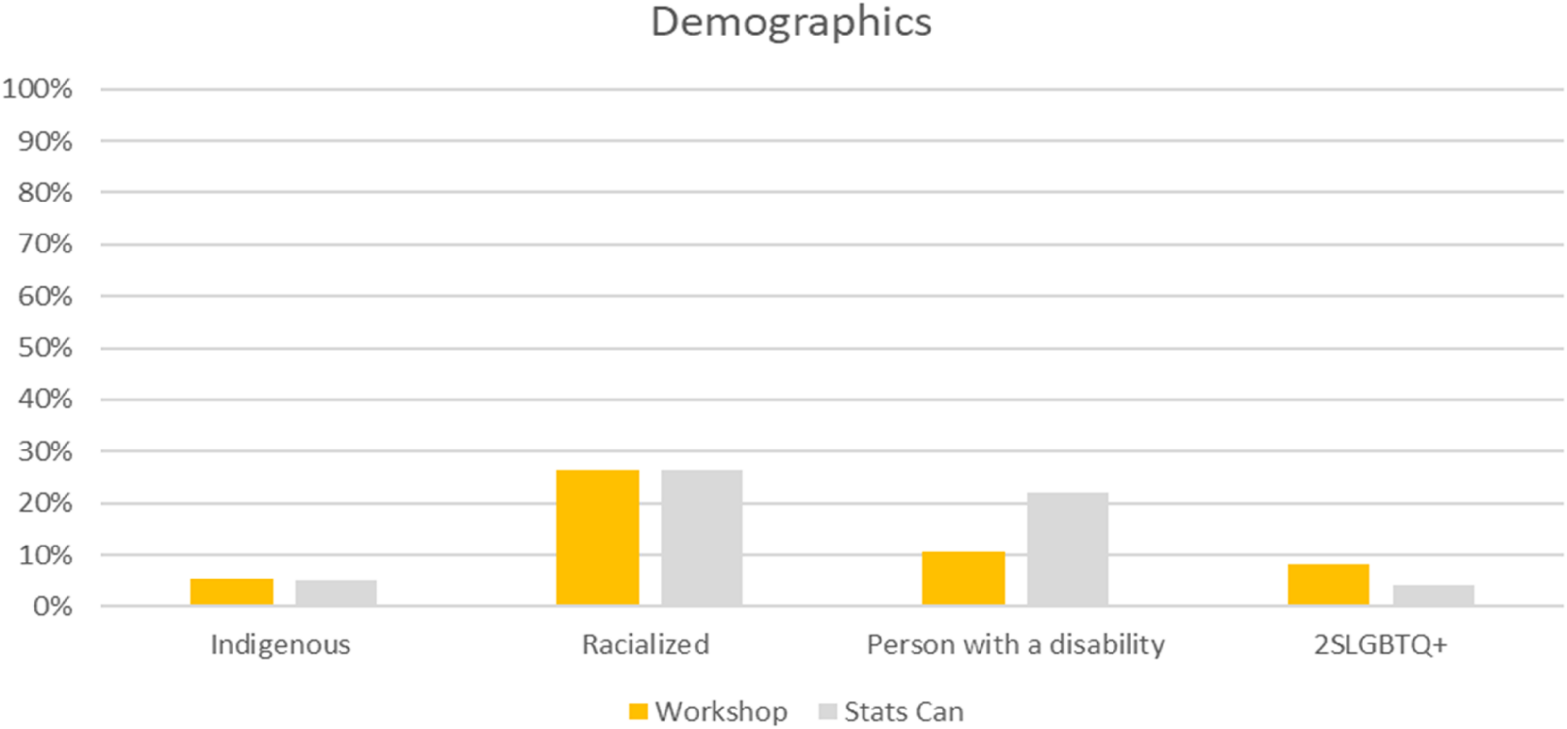
Comparison of workshop attendee demographics to statistics Canada population data.

Thirty-eight people attended the event. As a basic check on the representativeness of event attendees in relation to the diversity of the general Canadian population, we compared attendee demographics characteristics to Statistics Canada data (see Figure 1). The percentage of attendees who self-identified as Indigenous, racially and ethnically minoritized, or members of the 2SLGBTQI+ (Two-Spirit, Lesbian, Gay, Bisexual, Transgender, Queer and/or Questioning, Intersex, and many other ways people self-identify) community met or exceeded Stats Canada population figures, even when not including the five percent of attendees who indicated “preferred not to say” to each of those questions of identity. The workshop had an over-representation of women (66%) compared to the general population, though not inconsistent with healthcare gender disparities, where women make up 75% of the workforce (31). There were 11% of attendees who did not report or preferred not to provide information on their gender. Attendees who identified as a person with a disability (11%) were significantly underrepresented when compared to the general population (22%), though 13% of attendees did not provide a response to that question.

**Figure 2:**
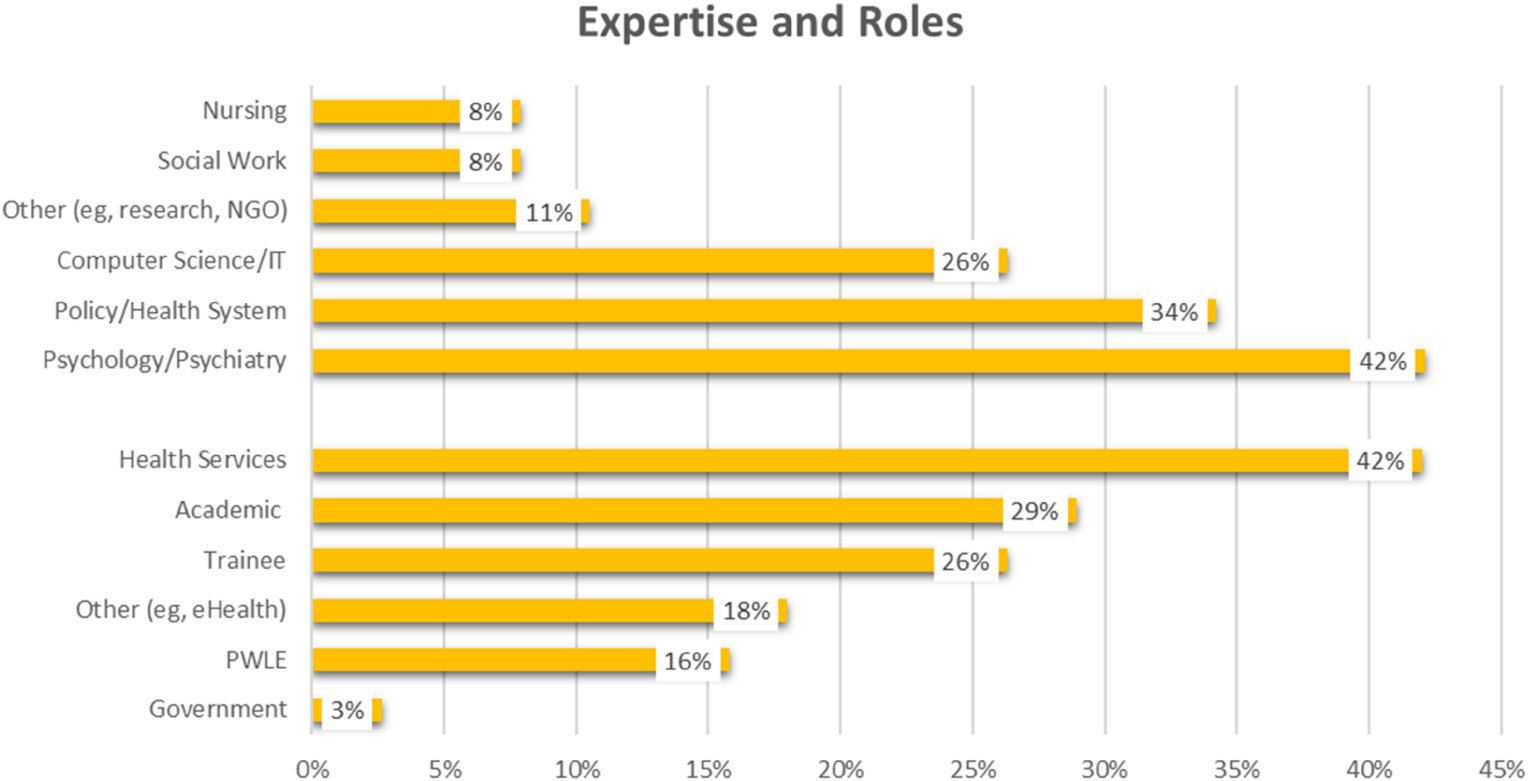
Percentage of attendees reporting disciplines of expertise and perspectives.

The group of attendees reflected diversity across disciplines and expertise (see Figure 2), including nursing (8%), social work (8%), computer science/information technologies (26%), policy/health system administration (34%) and psychology/psychiatry (42%). Reflective of the intersectional perspectives of each attendee, there were a mix of individuals with lived experience with mental health or substance use conditions (16%), trainees and students (26%), academic faculty (29%), and those in health service roles (e.g., clinicians, policymakers, administrators; 42%). A significant portion of the group (18%) identified “other” perspectives they brought to the discussion, including research staff, youth and caregiver advocates, Indigenous and EDI consultants, non-governmental organization/non-profit sector and digital service providers.

### 3.2 Challenges and action recommendations

The insights gathered from discussions groups brought to the forefront 11 key themes in relation to the challenges faced (see Table 1) and nine action recommendations to fortify the efficacy and inclusivity of digital health tools for youth in the future (see Table 2). Generally, racism, discrimination, and oppression were viewed as chronic and embedded in digital mental health innovation processes, including in the hierarchy and ownership of data, mental health knowledges, and promotional strategies involved in launching digital health services. The rapid rate of technology development means computer science and mental health-sector graduates must be prepared to address not only the challenges with current technologies, but also think critically about how their personal, academic, and professional environments will be shaped by digital health services in the future.

**Table 1:** Summary of themes emerging from discussion of challenges.

Themes	Selected examples of notes	Words in field notes
Digital mental health services perpetuating racism and white-colonial normativity	• Lack of representation in the content (stories, visuals, experiences) • Lack of representation in the clinicians and peers involved • Cultural stereotypes and stigmatizing language in digital service content • Limited language options for digital services • Historical mistrust of health system leads to help-seeking fear and avoidance of digital services • All -or -nothing systems that only value “experts” and don't train “lay people” • AI algorithms that are bias to white, cis-gendered behaviour • Decision-makers who are selecting and licensing these tools are not diverse/do not challenge racist and culturally unsafe services	Representation Distrust Systemic Racism Experts Algorithms Stereotypes Stigmatizing Decision-makers Unconscious bias
Lack of cultural humility in digital mental health research	• Lack of stewardship and self-governance for the people/communities expected to test and use these digital services • Ownership means Indigenous-led, otherwise it is just another colonial study • Need role models from racially and ethnically minoritized communities in digital mental health research • Lack of humility or cultural sensitivity in approaching equity-deserving populations about research and evaluation • Highly-structured research processes that leave no room for cultural nuances and limit interest and patience for research participation	Stewardship Ownership Highly-structured Colonization Disrespect Nuance Role models Trust-building Humility
Assumptions about “voice” during digital mental health community engagement	• Not engaging or inviting diverse voices or not making safe spaces for underrepresented people to share their input and experience • Youth 12 -26 are in very different stages but are often given same digital services • Assuming one “diverse” representative can speak for all/an entire community • People don't want to feel alone in their voice during engagement	Age range Voice Alone Engagement
Unequal access to fundamental tools for participating in a digital society	• Internet access is a human right • Affordability of devices is an issue for youth and families • Levels of government arguing over responsibility for digital infrastructure • Low digital literacy to even find, log-in, navigate services	Human rights Government Affordability Literacy
Lack of community-created and youth relevant digital mental health service promotion	• Digital services not promoted in the online spaces where youth go • Too much focus on social media promotion- need for peer-to peer endorsement and other elders/teachers/coaches/caring adults to promote • Promotional materials not created by youth • Promotional materials are generic and are not culturally relevant • Language of academics and bureaucrats and not the everyday language youth use among themselves • Fatigue with Zoom and digital “everything” all the time • Not great to reframe digital as “replacements” to in-person instead of it being one option	Generic Fatigue Word-of-mouth Social media Everyday Academics
Tension between healthy online and offline lives	• More digital services isn't necessarily good - loss of offline social connection with real people in community • Negative effect of some/too much technology on youth (social media) • Preferences for in-person care • Tech “addiction” • Tech feels impersonal and not one-on-one	Human connection Real-people Preferences Addiction Impersonal Replacement
Missing EDI measurement and evidence-base	• Not great evidence that they help/what they help • Unregulated and potentially unsafe • Clinician hesitation so as not to promote tools that are not evidence-based • Lack of EDI assessment of digital service content or features • Industry has no requirement for providing culturally responsive services • Digital tools can lead youth to mis/self-diagnosis • Evidence-based tools are not centralized/curated • Few guidelines or standards for EDI in digital health	Evidence Unregulated Hesitation Measurement Self-diagnosis Navigation
Funding structures that reward already privileged teams	• Digital mental health researchers are not funded compared to other health tech sectors • Digital mental health links two very different disciplines so funding and publication pathways can be harder • Community-members with innovative ideas have no access to funding outside of academia (which is predominantly white, economically well-off/high SES) • Participants in equity-deserving populations are not always well compensated (childcare, parking) or valued for their contributions to research	Underfunded Community-led Innovation Compensation
Disconnection and duplication of effort	• Federal and provincial services overlap and create redundancy • Continuum of services (when to use which service for what purpose) is not clear • There isn't one service or one option that is going to work for all • Digital mental health needs to be interwoven with other health • Lack of IT infrastructure to link population level digital mental health services to the “formal” system • Focused on crisis and treatment instead of prevention	Saturated Continuum Interwoven Infrastructure Systems Coordination Prevention
Gatekeeping that disempowers diverse youth	• Youth have to get permission to sign up or access services they need • Lack of privacy in using services or seeking help even digitally • Same people who access the system are the ones who find out about other digital tools and services – not reaching many youth • Different cultural and family values about mental health and mental health services • Families have fatigue over trying to find programs and digital services are not well explained to them	Consent Restriction Pathways Complex Values Culture
Digital design that isn't culturally informed or youth-friendly	• Limited use of tech functionality to personalize to individual needs, cultures, experiences • Overbuilt and clunky to navigate for many youth • Engagement with services is low but unclear why • Takes so long to demonstrate evidence that tech is outdated before its publicly available • Too focused on self-guided and self-help- not supportive enough for many youth • Few diverse youth engaged in testing and evaluating tools so developers are launching tools with very limited usability data	Personalization Tailored Engagement Safety Support

**Table 2:** Summary of themes emerging from discussion needed actions.

Themes	Selected examples of notes	Words in field notes
Stronger policies around EDRIA (equity, diversity, reconciliation, inclusivity, accessibility) can support accountability	• Review digital service content and processes for cultural safety before being promoted • Key success indicators should include dimensions of diversity • Align with best- practice and standards for AI to reduce bias • Signal to developers what is required so they build more diverse and inclusive apps/tools • Develop digital service standards and frameworks for evaluation • If you want something to be equitable you need to define what equitable means • Review current policies about promoting and licensing digital services and realign to evolving EDI understandings	Bias Reform Requirements Standards Evaluate Lens Anti-racist Competency Action
Funding needs to flow to community innovators not just senior researchers and in more creative ways	• Formal academic structures and traditional funding are inaccessible to many • Exploring funding partnerships (e.g., philanthropy, special cross-sector funding) • Get access to funding faster (instead of just on certain cycles/calls for proposals) • Incubator funding for digital mental health • Funding so new ideas don't stagnate- accelerate emerging ideas through evidence building phases more quickly • Funding to go to community-led innovation and digital mental health	Alternative Incubation Partnership Intersectional Nurture Quicker Evidence-based
Building access to tech infrastructure for all	• Investments in internet and tech in all communities (rural, northern, etc.) • Reframe and advocate for technology as a human right • Infrastructure for building digital mental health tech not just physical health • Getting digital mental health out of the hands of industry and built instead by community	Essential Rights Social determinants Infrastructure
Ensuring a trauma informed lens is brought to both health research AND tech development teams	• Commitment toward reconciliation and rebuilding trust that might take time • “Nothing about us without us” • Acquire deeper knowledge of community histories and local needs • Trauma informed approaches brought into research and digital intervention design/development not just for those delivering direct clinical care	History Community Balance Preference Trauma-informed Reconciliation Authentic
Investing in supports for continuous digital skill upgrading at all levels	• Tech support and troubleshooting • Navigating digital mental health services and tools • Clinicians need ongoing tech support to work with clients in using these services	Literacy Navigation Exposure
Creating spaces and places to **begin** partnerships with equity-deserving communities	• If relationships and trust take time then trainees, community and researchers need more spaces to connect, talk, learn from each other early and often- not just for research funding • Training junior researchers on how to approach communities and importance of reciprocity • Cultural partnerships are important but also with youth of different ages	Humility Partnership Co-design/create Relationship Trust Age-relevant
Working together at local, regional, national and global levels.	• Global user community has a lot of teach us about who and how different technologies have an impact • Don't reinvent the wheel – learn from the global community	Global National Linkage Inspire
Resourcing diverse youth to co-create and lead promotional efforts around digital health services	• Working with youth and people who have expertise in messaging and communication • Getting clearer on the benefits of digital mental health • Stories of lived experience and how tools made a real difference • Simplify where to go to find evidence based tools • Expand community outreach workers knowledge of digital mental health services	Expertise Marketing Stories Outreach Youth-led
Activating allyship across research, policy and practice	• Race-based data and evaluation of digital services led by community • Challenging tokenism in digital mental health service selection and avoiding filing cabinet reports • Acknowledgement and honesty from researchers • Pushing back against push back (e.g., when indigenous research methods in university courses are not viewed as credible) • “calling out” racist content and approaches • Holding people accountable to EDRI policy and commitments • Leaders making space and privileging equity-deserving voices • Requiring EDRI training and cultural safety education in undergrad	Race-based Calling-out Empower Truthful Amplify

### 3.3 Panel discussion

In responding to audience-questions, panelists highlighted challenges, successes, and lessons learned about diversity in digital mental health services. Specifically, (a) how transdisciplinary knowledge is vital to the future of digital health design and implementation but requires the time to build new shared language across disciplines, (b) how important it is for leaders to be curious and not avoid learning about emerging or unfamiliar technologies and ways they might address inequities; (c) how too often adult assumptions about what youth want guide digital health policy (e.g., believing all youth want to engage with digital mental health services); and (d) how culturally-safe care a low bar aimed at limiting harm but culturally-centered care prioritizes the intersection of mental health and culture and actively seeks to integrate cultural knowledge, awareness, and understanding into digital health service design.

### 3.4 Event evaluations

Fifty percent of attendees (19/38) completed post-event evaluations (see Table 3). Overall, attendees saw value in making intersectional and interdisciplinary connections with 100% of those who responded (*n* = 19) strongly agreeing or agreeing the event allowed them to engage others they otherwise would not have met. As one attendee commented:

“I owe the knowledge I’ve gained to attending the event. The table grouping was like magic, providing me with insights into existing digital tools from various angles. This experience allowed me to uncover numerous research avenues that have the potential to enhance our understanding and advance digital tools for mental health to a new level.” – Attendee

**Table 3:** Knowledge exchange event attendee evaluations.

	Strongly agree/agree *n* (%)	Strongly disagree/disagree *n* (%)
I have engaged with researchers, healthcare providers, decision-makers, and/or patients/citizens I otherwise would not have met.	19 (100%)	0 (0%)
I have a greater understanding of the research gaps and policy needs around inclusive and equitable digital mental health topics presented today.	18 (95%)	1 (5%)
	Extremely/very *n* (%)	Somewhat/not at all *n* (%)
How relevant was today's event to your professional work, training or personal areas of interest?	17 (90%)	2 (10%)

## 4 Recommendations

The knowledge exchange workshop is one of the first of its kind in Canada to bring transdisciplinary and cross-sector groups together to jointly share experience and discuss current and future tensions within the system of digital mental health services for youth populations specifically. The workshop built off of prior local focus groups and workshops with youth themselves and was only one component of a larger series of local and national youth engagement sessions designed to ensure youth perspectives are heard alongside other key partners on the issue. Results of those other engagements will be published elsewhere. While a number of attendees were youth (aged 26 and under) there is value in thinking about how future events could combine audiences in these kinds of workshops so that youth have direct contact with policy and health system leadership.

### 4.1 Policy

Most challenges and necessary actions raised by attendees were situated at the organizational and community levels. This should be a signal that while action to address digital health service inequities can be undertaken by individuals, explicit and deliberate action is required by governments, health care and academic institutions to facilitate larger impacts on structural factors. This finding aligns with recent American Psychological Association guidelines promoting population health approaches that leverage technology to promote community health not just promoting online tools to individuals (32). Further, findings from the workshop suggest broadening our understanding of the role of digital tools in prevention and early intervention to maintain wellbeing not just as interventional treatment for youth with significant symptoms or functional impairment. Funding sources and appropriate timelines are required from funding agencies to allow researchers and communities to build true and genuine relationships to build digital health tools for and with community Our findings support recommendations for increasing digital mental health reach and uptake through youth and community engagement (33). This will become increasingly important in Canada as the recently launched Mental Health Commission of Canada *eMental Health Strategy for Canada* has explicitly identified the need to address EDI gaps and to engage those with lived experience in co-identifying policy and service priorities (34). Once digital health tools have been developed, their release into the live environment of the healthcare system offers critical moments for evaluating and addressing any impacts on health inequity and health systems leaders should prioritize that evaluation. Policies should not only enhance access to digital infrastructure but prioritize digital mental health initiatives that genuinely reflect local cultural dynamics and youth needs. For example, educational messaging and promotion around digital mental health service benefits and relevance is best created by youth, for youth in their own communities (35). These policies should seamlessly blend technology innovation aims while prioritizing trust-building with diverse communities.

### 4.2 Training

To better prepare trainees to advocate for and create more inclusive and equitable mental health technologies they must be encouraged to interrogate both the systems and structures around them and the products and therapeutic innovations they create. Both computer science and mental health clinicians connected to digital mental health design (especially those from dominant identities based on race, ethnicity, gender, sexuality, ability, and socioeconomic class) should deepen their cultural competence. Training that validates, facilitates, liberates, and empowers those who are historically underrepresented to cultivate their identities and direct digital health innovation should be prioritized (36). Attendees affirmed that building more equitable digital services can only happen through collaborative work. That 100% (*n* = 19) of attendees who evaluated the workshop indicated they made connections at the event with people they otherwise never would have underlies the necessity of organizational leadership in being intentional in organizing ongoing opportunities for knowledge exchange. Steps to increase cross-talk between disparate disciplines, perspectives and cultures can decrease feelings of isolation, and promote cultural humility and reciprocity. Within the Canadian landscape the DIVERT mental health training platform has a significant role to play in promoting a national community of transdisciplinary mental health researchers and clinicians that will champion an inclusive and accessible mental health care system for youth. Findings of the workshop highlight how post-secondary training needs to not only focus on digital and clinical competency development but building capacity in allyship, anti-oppressive practice and community partnership (37).

### 4.3 Research

Researchers and those who fund innovation opportunities should (a) center equity in their teams and theoretical approaches, (b) prioritize examination of the intersectionality of various factors such as race, gender, socioeconomic status, and sexual orientation in the context of youth digital mental health use and outcomes (38), (c) focus on issues of digital literacy and engagement, (d) use “out to the box” funding approaches to accelerate availability of culturally relevant, community-led services, (e) explore methods that amplify perspectives, world views and needs of underserved populations, (f) ensure ethical approaches for collecting, stewarding and using digital health data are used, (g) advance co-design methods (39) and research processes that amplify youth perspectives and preferences and influence the development of programs and policies that affect their lives (6), and (h) think about ways to make the tools they develop more easily adapted to different communities or marginalized groups. Generally, our findings align with and extend evidence from reviews of existing evidence (40, 41) adding to a rich and evolving global literature in digital mental health.

## 5 Limitations

People living with a disability (i.e., physical, sensory, and cognitive) were statistically underrepresented in the attendee group when compared to the general population. There was some health sector diversity representation but no attendees from occupational recreational therapy. We explored themes based on facilitator notes not on the first-voice of attendees in the discussion which may have introduced bias. While the authorship team engaged in the broader data analysis represented diverse intersectional perspectives, and there were opportunities for member-checking we recognize that the positionality of the primary coder may have introduced unintended bias. The post-workshop survey was not completed by all attendees and could reflect bias toward positive responses.

## 6 Conclusions

Active and continuous collaboration among these disciplines and roles is vital to ensure cultural, structural, financial, geographic, and material differences that shape a young person's ability to encounter and engage with digital mental health are considered. We urge digital health developers and mental health leaders to consider all aspects of digital equity, and to come together routinely to revisit the lessons learned here so that we achieve a more equitable, fair, and just mental healthcare system that leads to better outcomes for all youth.
