# Supplementary material for: Centering equity, diversity, and inclusion in youth digital mental health: findings from a research, policy, and practice knowledge exchange workshop
Source: Front Digit Health. 2024 Oct 31;6:1449129. doi: 10.3389/fdgth.2024.1449129 (PMC11560888; doi:10.3389/fdgth.2024.1449129)

**13.0 Supplementary Material**

**Pitch Event Presentations**

**(Clinical Psychology PhD Student)**

The Impact of Diversity Factor on the Effectiveness of and eHealth Treatment for Sleep Difficulties in Children


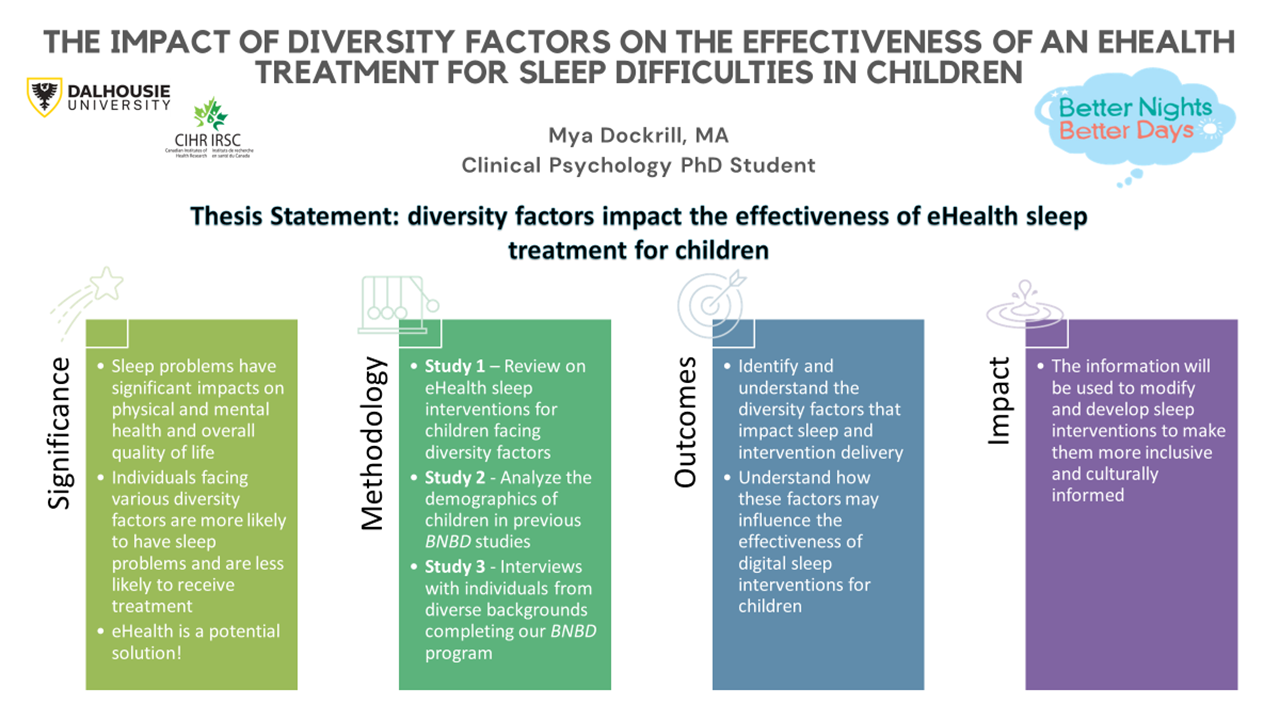


**(Department of Computer Science PhD Student)**

Personalized Mental Health Intervention for Attention Bias Modification Training (AMBT)


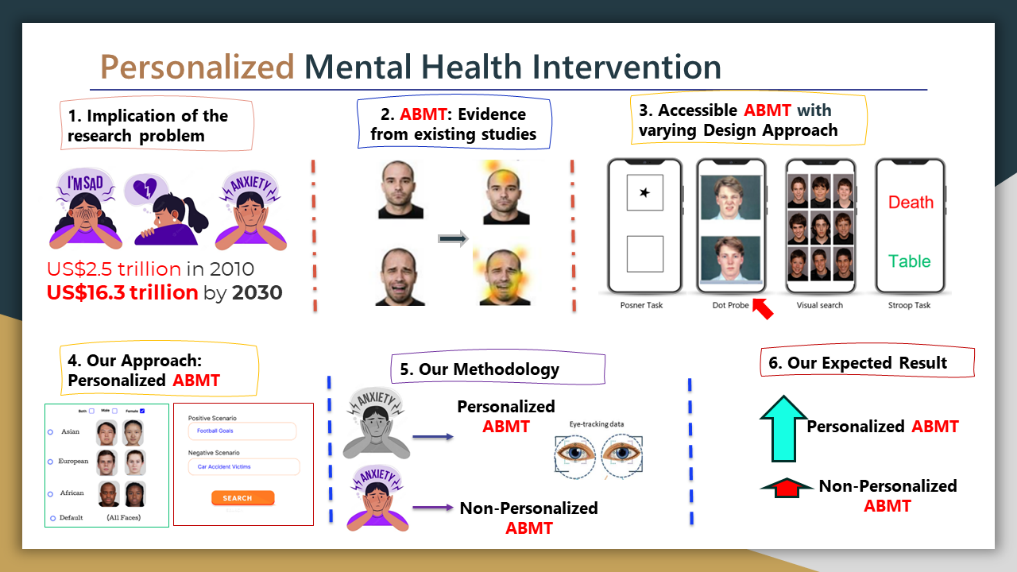


**(Department of Psychiatry PhD Candidate)**

Augmenting Mental Health Support for Patients Accessing Different Degrees of Formal Psychiatric Care Through a Supportive Text Messaging Program (Text4Support)


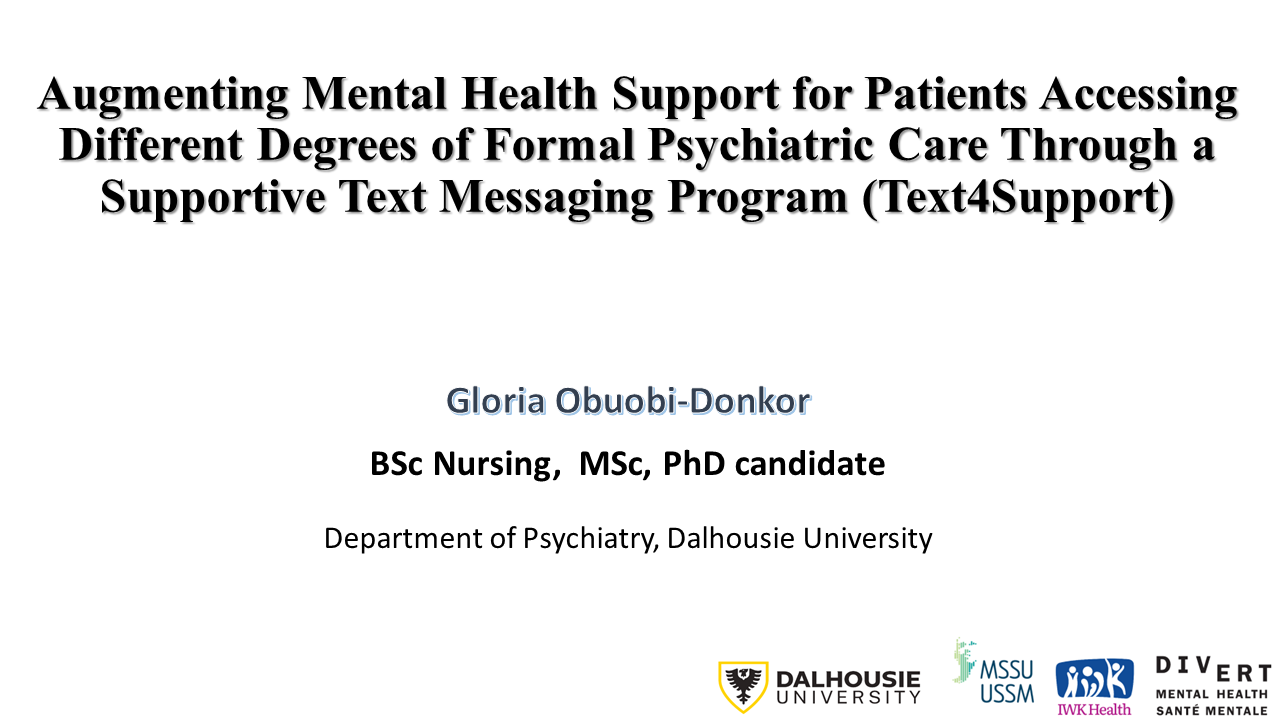

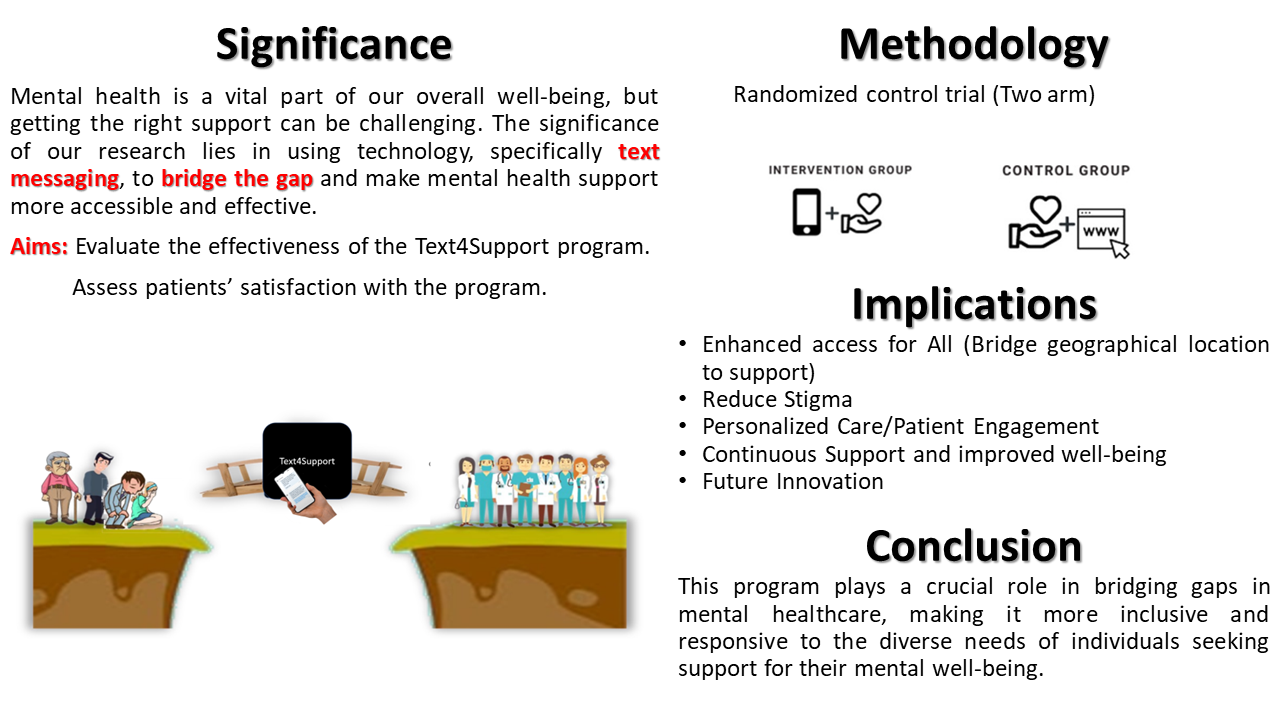


**(Clinical Psychology PhD Student)**

Addressing the sleep health of university students in Nova Scotia


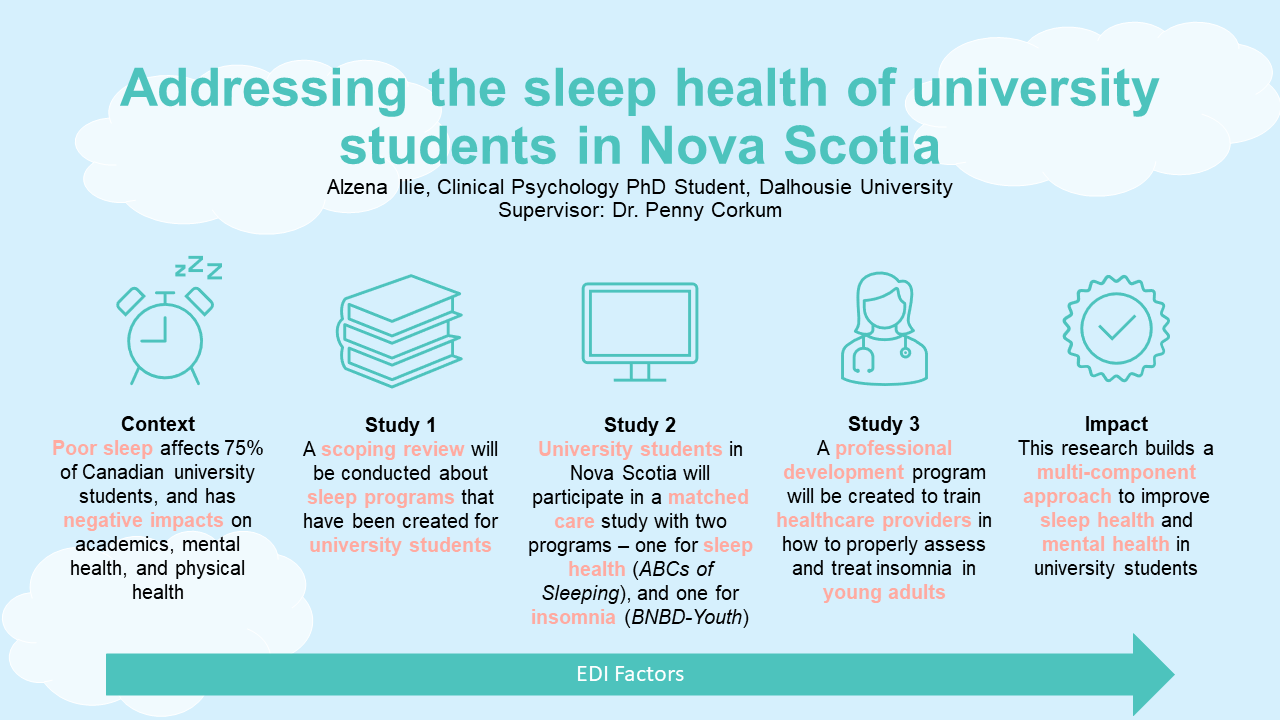


**(Psychology, BSc Honors Student)**

Paraprofessional coach- and telehealth delivered written exposure therapy for teen posttraumatic stress disorders


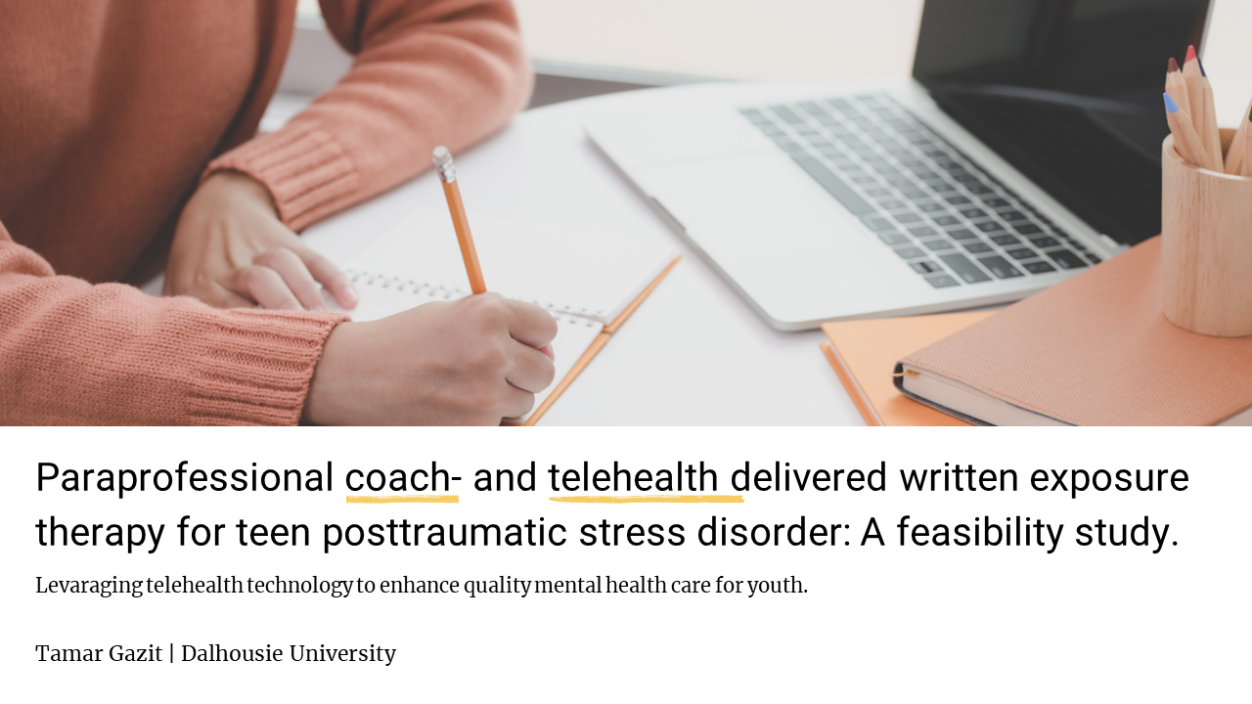


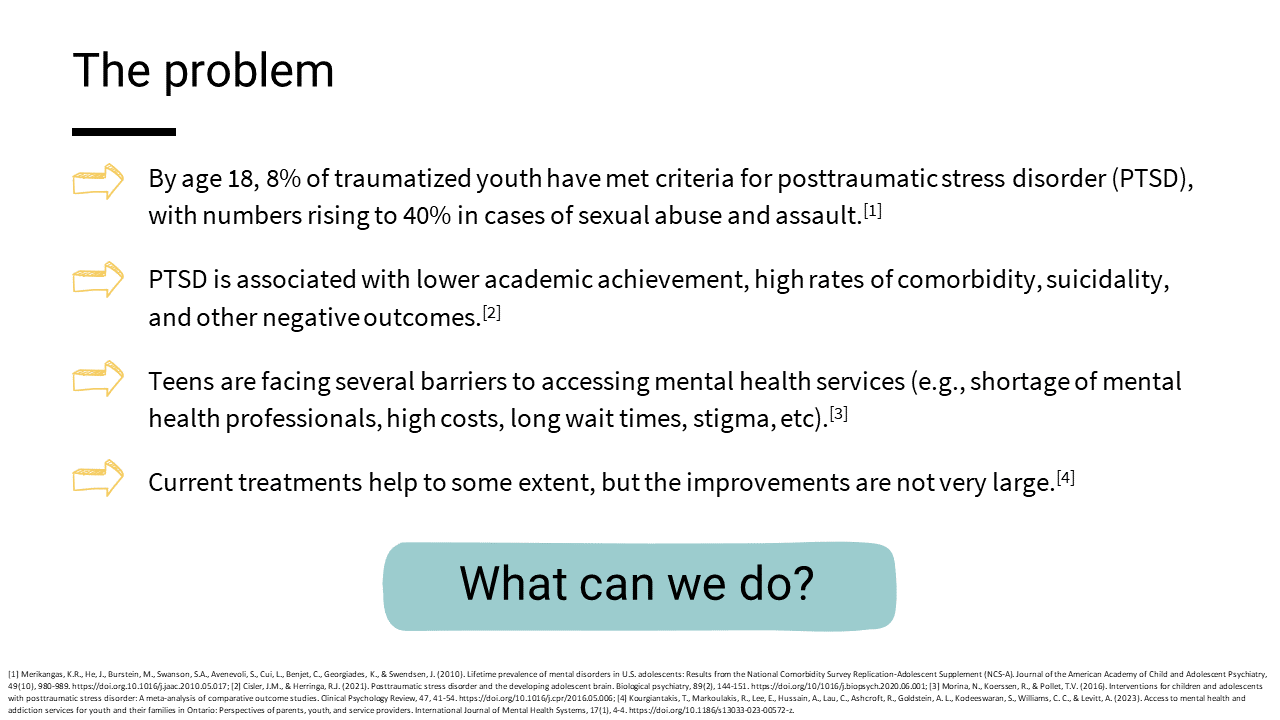


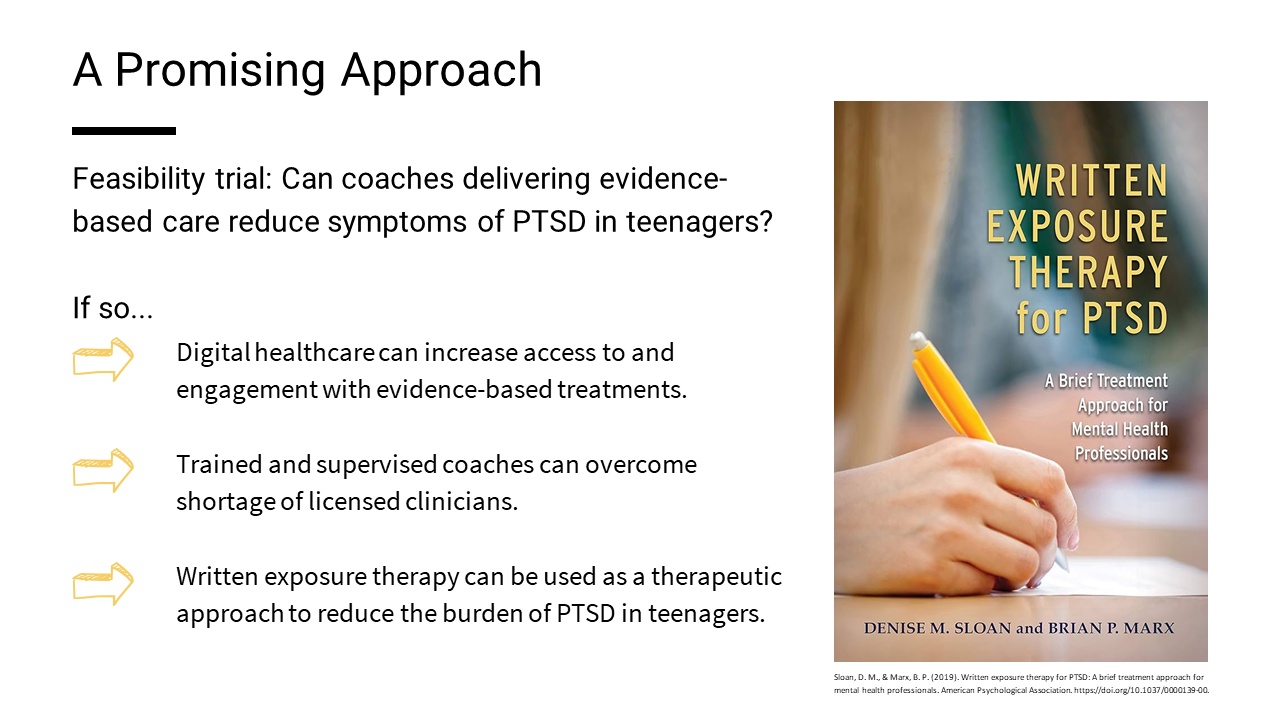

Supplement: Supplementary file 1 [file Datasheet1.docx]
